# Towards compact laser-driven accelerators: exploring the potential of advanced double-layer targets

**DOI:** 10.1140/epjti/s40485-023-00102-8

**Published:** 2023-06-08

**Authors:** Alessandro Maffini, Francesco Mirani, Marta Galbiati, Kevin Ambrogioni, Francesco Gatti, Maria Sole Galli De Magistris, Davide Vavassori, Davide Orecchia, David Dellasega, Valeria Russo, Margherita Zavelani-Rossi, Matteo Passoni

**Affiliations:** Dipartimento di Energia, Politecnico di Milano, Piazza L. Da Vinci, 32, Milano, 20133 Italy

**Keywords:** Laser-driven particle acceleration, Double-layer targets, Carbon foams, Pulsed-laser deposition, Magnetron sputtering, Particle induced X-ray emission

## Abstract

The interest in compact, cost-effective, and versatile accelerators is increasing for many applications of great societal relevance, ranging from nuclear medicine to agriculture, pollution control, and cultural heritage conservation. For instance, Particle Induced X-ray Emission (PIXE) is a non-destructive material characterization technique applied to environmental analysis that requires MeV-energy ions. In this context, superintense laser-driven ion sources represent a promising alternative to conventional accelerators. In particular, the optimization of the laser-target coupling by acting on target properties results in an enhancement of ion current and energy with reduced requirements on the laser system. Among the advanced target concepts that have been explored, one appealing option is given by double-layer targets (DLTs), where a very low-density layer, which acts as an enhanced laser absorber, is grown to a thin solid foil. Here we present some of the most recent results concerning the production with deposition techniques of advanced DLTs for laser-driven particle acceleration. We assess the potential of these targets for laser-driven ion acceleration with particle-in-cell simulations, as well as their application to PIXE analysis of aerosol samples with Monte Carlo simulations. Our investigation reports that MeV protons, accelerated with a ∼20 TW compact laser and optimized DLTs, can allow performing PIXE with comparable performances to conventional sources. We conclude that compact DLT-based laser-driven accelerators can be relevant for environmental monitoring.

## Introduction

Compact, flexible, and versatile ion and neutron sources are key for many scientific and technological applications of great societal relevance [1]. Laser-plasma-based ion acceleration is attracting growing interest as a promising solution to circumvent some limitations of conventional accelerators, such as non-tunable energy, high costs, non-portable size, and radioprotection issues. Generally speaking, a laser-driven accelerator of charged particles (e.g. electrons and ions) is based on the interaction of an ultra-intense ultra-short laser pulse ($\mathrm{I} > 10^{18}\text{ W}/\text{cm}^{2}$) with a target, which rapidly ionizes turning into a plasma. Focusing on ions, the coupling of the laser with the plasma induces a strong charge separation and, consequently, intense longitudinal electric fields which are responsible for the acceleration process [2]. Among the various laser-based ion acceleration mechanisms that have been proposed in the last two decades, the Target Normal Sheath Acceleration (TNSA) is one of the most reliable, robust, and understood. In TNSA, laser pulses are focused on a micrometric solid target and their energy is partially absorbed by the electrons of the target. Electrons are heated up to relativistic energies and expand towards the back side, generating a very intense longitudinal sheath electric field (few MV/μm). This field is responsible for the acceleration of the light ions (mostly protons) located on the rear surface of the target. The result is the emission of bunches of light ions (10^8^ up to 10^12^ protons per shot) with a broad energy spectrum (e.g. exponential distribution with an effective temperature in the order of few MeV) and a well-defined cut-off energy, ranging from few MeV up to several tens of MeV.

Thanks to their features, laser-driven ion sources are already of potential practical interest for some applications in the field of Ion Beam Analysis techniques, such as Particle Induced X-ray Emission (PIXE) [3–6]. PIXE is a non-destructive analytical technique for the elemental analysis of a large variety of materials (e.g. from cultural heritage to environmental samples) [7]. It relies on the interaction of MeV energy protons with materials having an unknown elemental composition and the detection of the emitted characteristic X-rays. Moreover, laser-driven ion beams can give rise to application-relevant secondary neutron sources by exploiting suitable converter materials, in the so-called pitcher-catcher scheme. However, to make laser-driven acceleration attractive for the most challenging applications (e.g. those requiring fast neutron [8] and high-energy photon generation [9]), an enhancement in acceleration performance in terms of energy and current of accelerated ions is required. A widely investigated approach relies on the continuous progress in laser technology along two main directions: multi-petawatt laser systems, characterized by high pulse energy (tens to hundreds of J) and low repetition rate (from few shots per minute down to few shots per day) and table-top lasers, with peak powers of tens to hundreds of terawatt (energy from tens of mJ up to few J) and a high repetition rate (from Hz up to kHz regime). The first direction ultimately relies on the availability of a limited number of top-class, state-of-the-art laser facilities. Therefore, it cannot find a widespread diffusion in developing countries. Whereas, the second class of lasers can lead to a practical, compact, and cost-effective alternative to conventional accelerators. This could become possible by adopting the strategy of enhancing the energy and number of accelerated ions via control and optimization of the laser-target coupling by acting on the target properties [10, 11]. In this respect, advanced Double-Layer Targets (DLTs), where a solid foil is covered with a low-density layer (e.g. near-critical carbon foams [12]), proved to be effective in increasing both maximum energy and the number of accelerated protons. Indeed, the laser pulse strongly interacts with the near-critical layer leading to a high conversion efficiency of laser-to-energy into the hot electrons responsible for the TNSA process [13]. DLTs can play a key role in achieving the proton energies and number required by several potential applications with compact laser systems.

In this work, we present a potential application having social relevance of laser-driven proton sources. To this aim, significant advances in the production of fully optimized DLTs are shown, and we discuss their potential use for laser-driven PIXE in the field of environmental analysis via numerical simulations. In Section 2 we present our strategies for the production of substrates and near-critical carbon foams by means of Physical Vapour Deposition (PVD) techniques such as Magnetron Sputtering and Pulsed-Laser Deposition (PLD), respectively. Then, the potentials of our targets for laser-driven particle acceleration are investigated with a broad campaign of Particle-In-Cell (PIC) simulations. At this stage, we consider laser parameters compatible with those achievable with a compact 20 TW system. Finally, we simulate an aerosol sample irradiation relevant to environmental analysis. The goal is to address the possibility of effectively performing laser-driven PIXE with our targets and table-top commercial lasers. This is achieved by providing the simulated proton energy spectra retrieved from PIC simulations to PIXE Monte Carlo simulations.

## Production of double-layer targets

The production of DLTs for laser-driven ion acceleration, attractive for multiple applications [4, 8, 10], requires a fine control of the properties of both the near-critical film and the solid substrate. These are, for instance, density and thickness, but also the cohesion between the two layers and the overall integrity of the component.

Our approach to developing advanced DLTs combines deposition techniques to achieve the production of the entire target on a suitable perforated holder. In previous works we have addressed in detail the fabrication of near-critical layers in the form of nanostructured carbon foams exploiting the PLD technique [12, 14, 15]. In this section, we will mostly focus on solid-density substrate manufacturing.

In typical TNSA experiments, rolled commercial sheets with nominal thickness spanning from 100 s of nanometers up to several microns are frequently employed [16]. These foils are obtained through mechanical processing of bulk materials, and therefore are mostly metallic, with limited availability in terms of elemental composition and thickness. Moreover, they can be characterized by tolerances of $\pm 30\%$ with respect to the nominal thickness values, as reported by manufacturers, and the presence of defects like pinholes and ripples is common. These sources of uncertainty affect the shot-to-shot reproducibility of laser-driven ion sources.

A promising alternative to commercially available rolled foils is the growth of free-standing films by means of PVD techniques since the versatility offered by PVD techniques can be exploited to tune the elemental composition, thickness, and morphology of the deposited films down to the nanoscale. In fact, it has been shown—both numerically and experimentally [17–19]—that target thickness greatly affects the characteristics of the accelerated ions, particularly in terms of their maximum energy. In addition, the possibility of using different materials is also valuable in some applications, in which the characteristic X-ray emission of the target material is used to irradiate samples and probe their composition [20].

In our study, substrates are deposited via Magnetron Sputtering technique [21], which allows depositing films from a few nanometers up to several microns tuning density, morphology, and stoichiometry with good uniformity over large areas (up to several cm^2^) [22]. Magnetron Sputtering is based on the application of a voltage between two electrodes in a working gas atmosphere, triggering the formation of a plasma. A magnetic field is applied at the cathode, confining the plasma and enhancing the sputtering by gas ions of the material to be deposited [23]. This technique can be operated as DC (constant applied voltage) or as HiPIMS (pulsed applied voltage). In general, DCMS films deposited at room temperature are characterized by a columnar morphology, while HiPIMS films exhibit a more compact structure.

The strategy that we proposed in a previous work [10] exploits the filling of the perforated holes of the target holder with a sacrificial material. In particular, sucrose showed to be a good candidate as it possesses high solubility in water, fast solidification, and low viscosity when dissolved. Practically, the holes are filled with a sucrose solution which then solidifies, leaving a continuous surface, on top of which the optimized solid film can be grown and, lastly, the sucrose is removed by soaking the holder in water.

**Figure 1:**
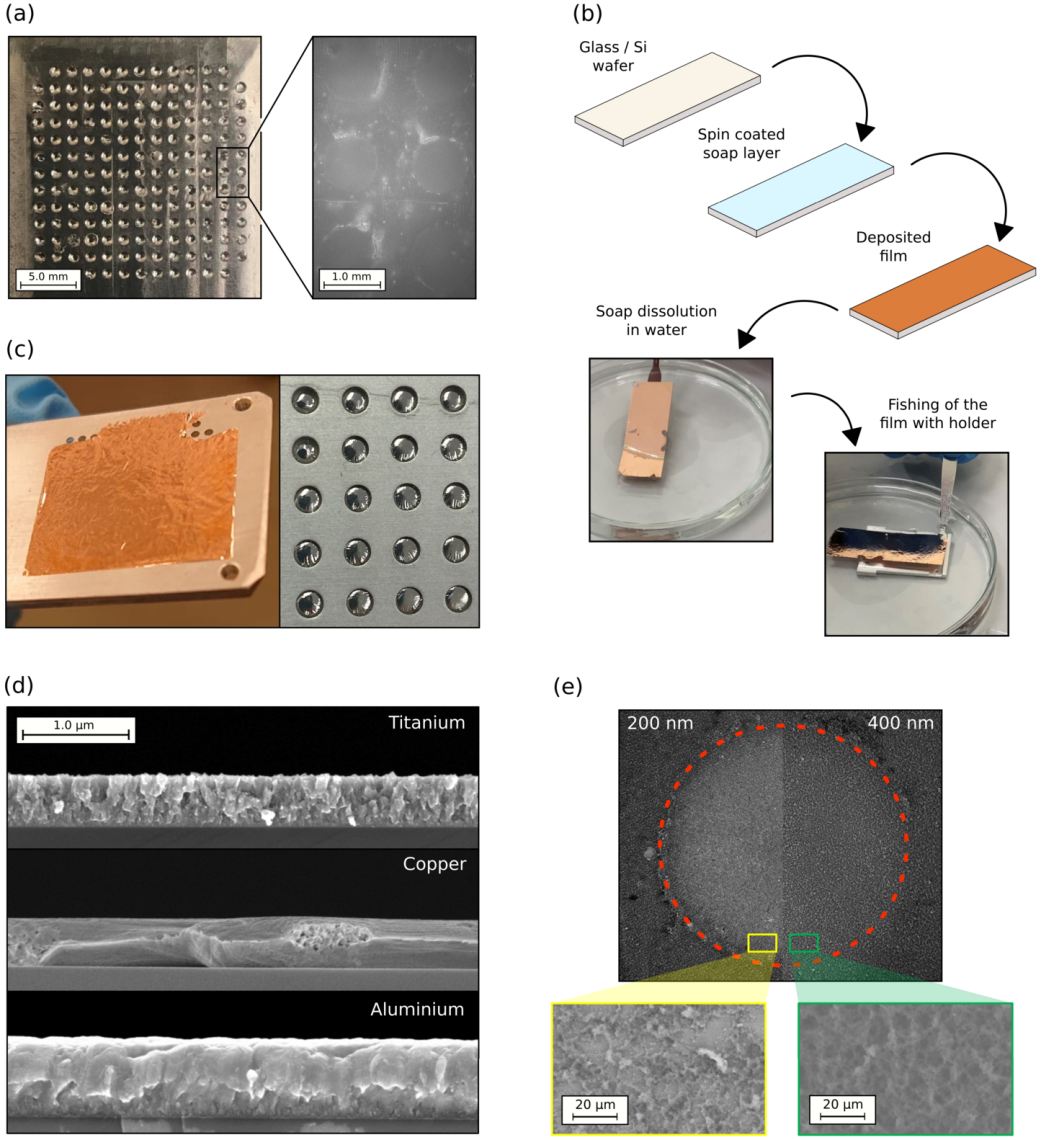
(**a**) Perforated target holder (1 mm holes) with free-standing titanium film and its magnification, showing the high planarity of the substrates; (**b**) scheme of the *fishing* procedure and pictures of a floating 200 nm copper film; (**c**) 200 nm copper (left) and 50 nm aluminum (right) films laying down on the target holders; (**d**) SEM cross section images of hybrid DCMS and HiPIMS titanium, DCMS copper and biased (100 V) HiPIMS aluminium (from up to bottom); (**e**) side-by-side comparison of the effect of different titanium substrate thickness (200 nm and 400 nm, reported on top) on low-density carbon foam deposition, with magnifications

The material we investigated mostly with this strategy is titanium (Ti). It has been chosen as it is a common substrate material in laser-driven ion acceleration experiments [24] and is characterized by good mechanical and thermal properties. The Ti layer is grown alternating DCMS and HiPIMS depositions to better control its morphology, density, and stress state. As a result, compact, quasi-amorphous substrates with thicknesses ranging from 200 nm up to 2 μm have been produced with maximum measured thickness uncertainty of $\pm 5\%$ over an area of several cm^2^ and a density greater than 80% of the bulk value. Figure 1(a) shows the target holder with the titanium target and a magnification of the free-standing region on the perforations.

Here, we present a second procedure, schematized in Fig. 1(b), based on a *fishing* approach [25, 26]. In this case, a silicon wafer or a glass slide is used as a supporting substrate during the deposition, over which a thin layer of commercial soap is spread by spin coating. So, after the deposition has been concluded, water can be used to detach the metallic film from the substrate. The film is left floating thanks to surface tension, which also helps in keeping it plane. Lastly, it can be scooped, or *fished*, directly on the target holder.

This production strategy works better with particularly ductile materials so that the films can detach without damage. In particular, we mainly investigated copper (Cu) and aluminium (Al), which are also among the most commonly employed materials for laser-driven ion acceleration experiments [2]. Considering Cu, we have been able to produce free-standing targets with thicknesses ranging from 50 nm to 1.8 μm, low stress and bulk density exploiting DCMS. As far as Al is concerned, depositions allowed us to produce substrates of thicknesses from 50 nm to 1 μm. A 200 nm Cu and 50 nm Al targets are visible in the left and right panels of Fig. 1(c) respectively. These films were produced through DCMS and HiPIMS.

The time needed for the production of the substrates depends on the specific material and deposition parameters. For instance, the growth rate of the optimized titanium films, which exploits a combination of DCMS and HiPIMS, is around 8.7 nm/min. On the other hand, for pure DCMS copper, it is around 65 nm/min. The deposition conditions must be carefully selected according to the specific material deposited. Limiting the discussion to the mentioned materials, Ti HiPIMS films, while showing a compact structure, exhibit also a highly compressive stress state which can result in damage to the sacrificial layer prior to its dissolution in water. On the other hand, DCMS Ti films show tensile stresses and columnar, poorly compact morphology. Thus, combining the two sputtering modes in a multilayer structure allowed the production of good DLT substrates. As far as Cu is concerned, DCMS mode provided satisfactory results in terms of compactness and stress state, granting the direct application of the ‘fishing’ strategy. Instead, the HiPIMS mode produced highly compressive stresses in the films, causing their curling once free-standing. Lastly, Al samples showed good results in DCMS and HiPIMS modes, both in terms of stress and morphology, giving flexibility on deposition conditions. Cross sections of all the three discussed films are reported in Fig. 1(d), where the difference between ductile Cu and Al compared to Ti is clearly appreciable.

Considering the two metallic substrate production strategies outlined here, few comparisons can be done. The hole-filling procedure adopted for titanium provides samples directly attached to the grid of the target holder. Thus, no wrinkles can form during manipulation, granting a plane surface which speeds up the focusing procedure during laser-target interaction experiments. However, this procedure is time-consuming since each hole must be filled individually. On the other hand, the *fishing* strategy is significantly simpler and faster. Though, the positioning of the film in the holder must be performed carefully to avoid creasing. Lastly, as reported previously, considerably thinner substrates were obtained by exploiting this second approach.

As anticipated, an important characteristic that needs to be evaluated to produce DLTs is the compatibility between the metallic layer and the low-density foam. In this regard, considering titanium films as a reference, it has been observed that for free-standing metallic film thicknesses below 400 nm, the deposition of the low-density layer is less effective. The total amount of foam material is reduced with respect to the other investigated cases, showing also a radial decrease from the periphery to the centre, and the microstructure is varied (see Fig. 1(e)). This could suggest that the vibrational solicitation of the film due to the impinging clusters during deposition prevents the further adhesion of aggregates and/or facilitate their partial detachment. As in elastic membrane vibrations, a mass dependence (and thus thickness, given the same density and area) of this phenomenon is foreseen.

## Particle-in-cell simulations of laser interaction with double-layer targets

We prove the feasibility and advantages of exploiting laser-DLT interaction for ion acceleration with state-of-the-art numerical simulations. PIC codes are the most established tools to simulate the interaction of ultra-intense femtosecond pulses with matter in the plasma state. Therefore, to investigate the most efficient conditions for DLT-based ion acceleration, we performed a scan of 2D simulations and a realistic 3D simulation using the highly-parallel PIC codes WarpX [27] and Smilei [28].

For the 2D numerical investigation with the GPU-accelerated code WarpX, parameters representative of a standard and compact but highly-focused laser source are chosen: peak power of 20 TW, intensity FWHM of 30 fs, wavelength of 0.8 μm and focal spot radius of 2.4 μm, resulting in an intensity of $\mathrm{I}\sim 2.2\cdot 10^{20}\text{ W}/\text{cm}^{2}$. The simulation box has dimensions $x\times y=100$ μm×56 μm, and laser propagation is parallel to the *x* direction. The spatial resolution is set to 65 points per μm. The simulation duration is 500 fs. The simulated target is inspired by the results described in Sect. 2 and is positioned inside the box at two times the distance the laser travels in its field FWHM. The target consists of a completely ionized homogeneous carbon foam with an electron density $n_{e}=2.6 n_{c}$ attached to a completely ionized aluminum foil with $n_{e}=450 n_{c}$ ($n_{c}= m_{e} \omega _{0}^{2}\epsilon _{0}/e^{2}$ is the critical density, $m_{e}$ the electron mass, $\omega _{0}$ the laser frequency, $\epsilon _{0}$ the vacuum permittivity and *e* the electron charge). A 0.05 μm-thick ionized hydrogen layer with $n_{e}=10 n_{c}$ is added on the rear surface of the substrate to simulate contaminants. The contaminant layer is the source of protons on which TNSA is most effective. We will focus on protons in the analysis of the results. The target electron distribution is sampled with 30 particles per cell (ppc) for substrate, 10 ppc for foam, and 100 ppc for contaminants, while ion distribution is sampled with 3 ppc, 1 ppc, and 100 ppc for substrate, foam, and contaminants respectively. The foam density is chosen according to the model exposed in [13]. This model predicts an optimal density and an optimal thickness to maximize the proton maximum energy once fixed the laser parameters. The thickness of both substrate and near-critical layer varies in the simulation scan. Following the results achieved in target production, we explore substrate thicknesses of 200 nm, 600 nm, and 2 μm. Each of these cases is simulated with four different foam thickness conditions: 0 μm (no foam), 2 μm, 4 μm (optimal thickness), and 8 μm.

**Figure 2:**
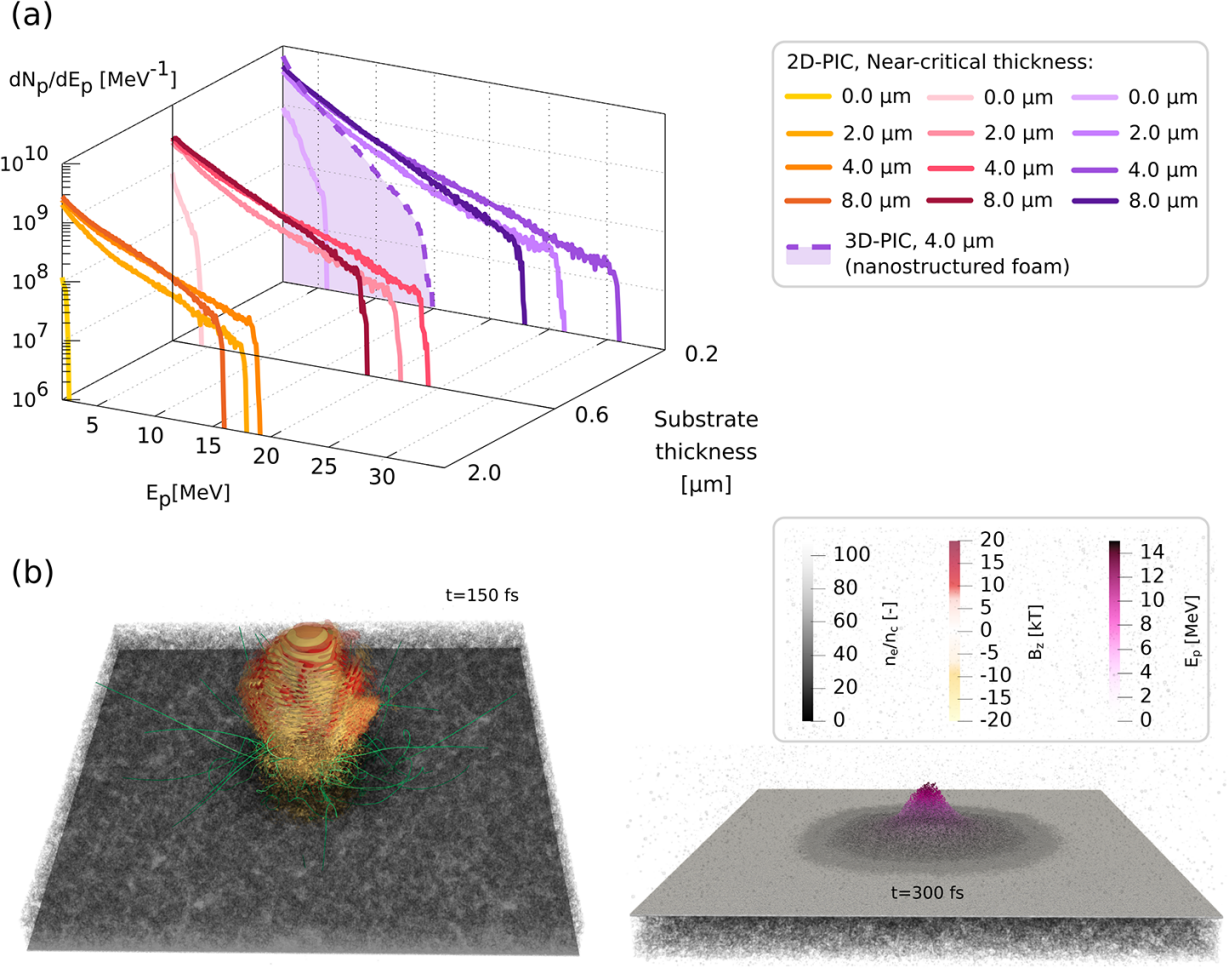
Some results of the PIC simulations. (**a**) shows the proton spectra normalized to give an approximated estimate of the experimental values in the 2D simulation scan (time selection is explained in Sect. 3) and at 300 fs in the 3D simulation. (**b**) shows two snapshots of the 3D simulation: in greys, the electron density normalized to $n_{c}$. At 150 fs the $B_{z}$ field and some foam electron trajectories in green are visible. At 300 fs the accelerated protons are represented as spheres colored according to their energy $E_{p}$

While allowing performing large simulation scans for their reduced computational costs, 2D simulations provide less realistic analysis compared to full-dimensionality investigations. Some physical details of the accelerating process are altered in 2D configuration due to constraints on particle motion. Enhanced re-circulation effects and alteration of the sheath electric field have been documented [29, 30]. In the 2D case, protons feel a nearly logarithmic scalar potential [31], and, consequently, for long times their energy monotonically increases without reaching a plateau [32] contrary to the 3D case. We use the strategy of [33] to have a unique choice of simulation time in which extracting relevant quantitative information on protons, i.e. their spectra (Fig. 2(a)), average and maximum energy ($\bar{E}_{p}$ and $E_{p}^{\mathrm{max}}$), temperature $T_{p}$, and efficiency *η* of energy conversion from the laser (see Table 1). The strategy for time selection is the following. We fitted the evolution of the maximum proton energy of the bare substrate cases with a logarithmic shape $E= E_{\infty }\log (t/t^{*})$ according to the electrostatic model of [33] to retrieve $E_{\infty}$ which should match the final maximum proton energy achieved in 3D. The time interval between the starting of proton acceleration and the time at which $E_{\infty}$ is reached in these cases is used to select the time after the starting of the proton acceleration at which we extract the mentioned quantities in the corresponding cases with DLTs. The logarithmic model is not directly applied to the double-layer target scheme because the enhanced proton acceleration process enlarges the transient time in which the electrostatic model is not valid, thus reducing the applicability of the fitting.

**Table 1:** Quantities related to protons obtained in the 2D simulation scan (time selection is explained in Sect. 3) changing foam and substrate thicknesses and in the 3D simulation with nanostructured foam at 300 fs. The reported quantities are the maximum energy $E_{p}^{\mathrm{max}}$ reached by the protons, their mean energy $\bar{E}_{p}$ above 0.5 MeV, their temperature $T_{p}$ coming from the exponential fitting of the spectra, the conversion efficiency *η* from laser energy to proton energy, and the estimate $N_{p}$ of the total number of accelerated protons per shot above 0.5 MeV

Foam thickness	$E_{p}^{\mathrm{max}}$ (MeV)	$\bar{E}_{p}$ [MeV]	$T_{p}$ [MeV]	Efficiency *η* [-]	N_*p*_ [-]
Substrate Thickness = 0.2 μm					
No foam	5.86	1.32	1.09	0.52%	5.12⋅10^9^
2 μm	26.24	3.44	5.03	5.48%	2.07⋅10^10^
4 μm	31.05	4.2	6.04	8.31%	2.44⋅10^10^
8 μm	22.83	3.88	4.07	7.28%	2.57⋅10^10^
Substrate Thickness = 0.6 μm					
No foam	4.56	1.26	1.03	0.25%	2.58⋅10^9^
2 μm	21.63	3.55	5.54	3.64%	1.33⋅10^10^
4 μm	24.04	4.41	5.91	5.72%	1.76⋅10^10^
8 μm	18.73	3.93	4.16	5.33%	1.69⋅10^10^
Substrate Thickness = 2 μm					
No foam	2.69	0.91	0.61	0.07%	1.0⋅10^9^
2 μm	17.93	3.27	4.13	2.61%	1.04⋅10^10^
4 μm	19.03	4.02	5.24	4.58%	1.63⋅10^10^
8 μm	15.92	3.60	3.62	4.50%	1.48⋅10^10^
Nanostructured 3D (Substrate Thickness = 0.2 μm)					
4 μm	15.05	1.85	2.20	4.58%	4.08⋅10^10^

Given the conversion efficiencies *η* from laser energy to proton energy, we give an estimate of the number of protons achievable in experimental conditions. From published data [34], we estimate the number of emitted protons in the forward direction for the bare 2 μm-thick target condition as $N_{p, 0} = 10^{9}$ $\mathrm{protons/shot}$. Then, we retrieve the number of accelerated protons $N_{p,i}$ for the other foam-attached cases. The laser energy $E_{L}$ transferred to the proton population can be expressed as $\eta _{i} E_{L} = N_{p,i} \bar{E}_{p,i}$, where the conversion efficiencies $\eta _{i}$ and mean proton energies $\bar{E}_{p,i}$ are retrieved from the PIC simulations considering proton energies $E_{p} > 0.5\text{ MeV}$. Since the laser energy is the same for all target configurations, we can scale up the accelerated protons per shot for all the target configurations as:

1 $$ N_{p,i} = \frac{\eta _{i}}{\eta _{0}} \frac{\bar{E}_{p,0}}{\bar{E}_{p,i}}N_{p,0}, $$

where $\eta _{0}$ is the conversion efficiency for the bare 2 μm-thick target condition. The estimated values of $N_{p}$ are reported in the last column of Table 1.

Figure 2 (a) shows the proton spectra for energies above 0.5 MeV obtained in the simulation scan normalized to have areas corresponding to $N_{p,i}$. All the spectra have an exponential shape with a cut-off typical of TNSA. The inclusion of the foam clearly enhances the energy and number of accelerated protons with respect to the bare substrate cases. This effect is mainly due to the reduction of reflection and the enhanced absorption of laser energy. A population of hotter electrons is generated which can accelerate the protons more efficiently. These considerations are validated by the enhancement of all the proton quantities reported in Table 1 in the foam-attached cases. Due to enhanced electron re-circulation along the substrate, the same quantities are increased by decreasing substrate thickness, a known fact in TNSA [35, 36]. In particular, overall maximum values are reached for the optimal foam length, confirming the results of the model in [13].

While the 2D scan allows verifying the most efficient conditions for proton acceleration, i.e. thin substrate and optimal density and foam, the retrieved values must be cautiously used due to the mentioned limitations of 2D simulations. Therefore, we performed a 3D simulation for the optimal case of 0.2 μm substrate and 4 μm foam. With respect to the 2D case, we updated the simulation setup for some parameters to reduce the computational cost but still deliver an accurate simulation. The 3D box is $x\times y\times z=70$ μm×50 μm×50 μm with 25 cells per μm. The simulation duration is 300 fs. For accurate target simulation, we included the three-dimensional nanostructure morphology simulated according to the Diffusion Limited Cluster-Cluster Aggregation (DLCCA) model [37]. Indeed, when laser intensities are not high enough, the nanostructure resulting from the foam growth process persists several times after laser interaction with foam itself [38]. The morphology consists of clusters of nanoparticles of radius 40 nm, density 20.78$n_{c}$ aggregated with a filling factor of 12.5% to generate a mean foam density of 2.6$n_{c}$. We performed this simulation with the PIC code Smilei because, despite not being yet GPU-accelerated, it allows easy inclusion of complex target morphology through the definition of arbitrary densities. In this 3D case, the target electron distribution is sampled with 10 particles per cell (ppc) for the substrate simulated with a density $n_{e}=80 n_{c}$, 20 ppc for foam, and 60 ppc for contaminants, while ion density is sampled with 1 ppc, 2 ppc, and 60 ppc for substrate, foam, and contaminants respectively. Figure 2(a) reports also the proton spectrum of the 3D simulation at 300 fs when maximum proton energy has already been stable for some timesteps. Figure 2(b) reports two snapshots of the simulation: at 150 fs when on the front side of the target the impinging laser pulse and some foam electron trajectories in green are visible and at 300 fs when the accelerated protons are visible on the rear side of the target as spheres colored according to their energy. The same quantities analyzed in the 2D simulations are reported for the 3D case in Table 1. Laser absorption is greater in disorderly nanostructured foams than in uniform ones and conversion efficiency into ion kinetic energy is increased by the explosion of the nanostructures [37]. However, the density inhomogeneities cause chaotic motion of electrons which alter the resonances inducing the direct laser acceleration process: maximum electron energy reduces while low energy electrons increase in number. This fact directly impacts the proton acceleration leading to lower maximum energy $E_{p}^{\mathrm{max}}$ of protons but relatively high conversion efficiency *η* (see Table 1).

## Simulation of laser-driven particle induced X-ray emission for environmental analysis

To evaluate the performances of laser-driven PIXE in the field of environmental analysis, we performed a broad campaign of Geant4 [39] Monte Carlo (MC) simulations of an aerosol sample irradiation [40–42] with different sources. The goal is to compare the number of emitted characteristic X-rays per unit time $\dot{N}_{x}$ from the interaction of laser-driven and monoenergetic protons (e.g. provided by a Van de Graaff or Tandem machine) with the sample. We performed 3 PIXE simulations considering monoenergetic protons of energies equal to 2.5, 4.0, and 5.5 MeV. Moreover, we carried out 12 MC simulations of laser-driven PIXE, exploiting the results obtained from 2D-PIC simulations. Even if 2D-PIC simulations are subject to a certain degree of inaccuracy as discussed in the previous section, this scan can provide preliminary outcomes to compare laser-driven and monoenergetic sources for PIXE environment analysis.

For laser-driven PIXE, the primary proton energies are sampled, between 0.5 MeV and the cut-off energies, from the spectra presented in Fig. 2(b). For each monoenergetic and laser-driven proton source, 3−10 ⋅10^8^ primary protons have been simulated. In all configurations, the irradiated material composition is representative of samples exploited for atmospheric aerosol analysis (NIST-1832). It is a disk having 150 μm thickness, a diameter of 5 cm and 0.384 g/cm^3^ density. Its main constituent is nitrocellulose which is composed of low-Z elements. The goal of the analysis is to identify the heavier elements present in low concentrations (see Table 2).

**Table 2:** Mass concentrations in % of the elements in the simulated aerosol sample

Nitrocellulose	Ca	V	Mn	Fe	Co	Cu	Pb
99.32909	0.38226	0.08776	0.08776	0.00061	0.01892	0.04680	0.04680

Protons are generated 15 cm far from the sample in vacuum. We neglect the angular divergence of laser-driven protons since the sample is large enough so that almost all primary particles interact with the material. Multiple scattering, ionization, and bremsstrahlung emission are taken into account for charged particles, while pair production, Compton scattering, and photoelectric effect are activated for photons with the G4EmStandardPhysics_option3 module. The production cuts for secondary particles are set equal to 5 μm. We select the ECPSSR model for the ionization cross sections [43]. The energies of the emitted X-rays from the sample are smeared according to a Gaussian distribution with FWHM $= 0.15\text{ keV}$ to take into account the energy resolution of detection systems. Lastly, the energies are collected in spectra.

**Figure 3:**
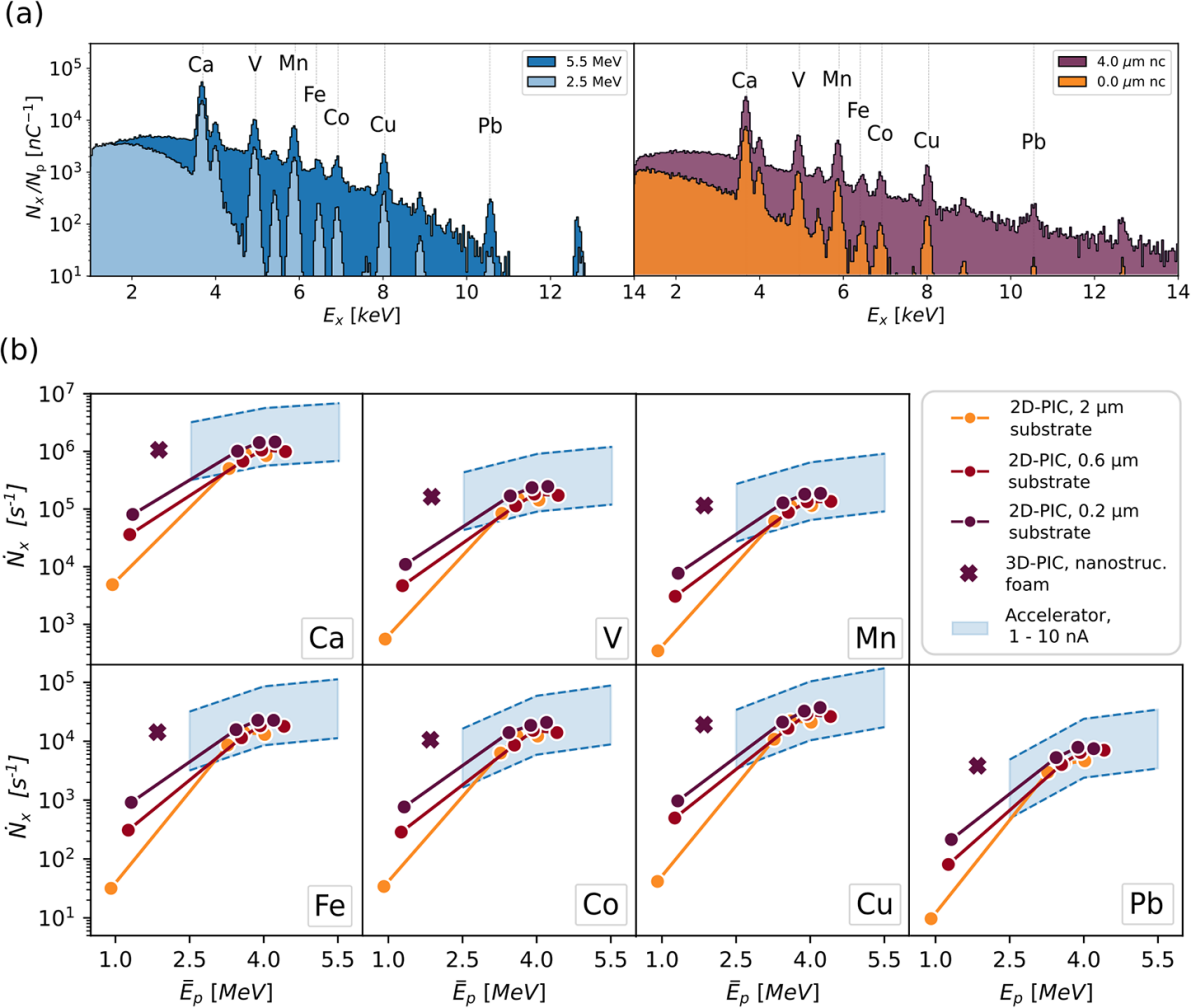
(**a**) Simulated X-ray energy spectra emitted from the sample and normalized to the incident proton charge. The left spectra are obtained with 2.5 and 5.5 MeV protons; the right spectra result from 2D-PIC laser-driven proton irradiation (200 nm substrate with 0 and 4 μm near-critical thickness case studies). (**b**) Number of emitted characteristic X-rays per unit time as a function of the average incident proton energy for all the elements. Blue color bands refer to conventional accelerators; purple, red and yellow round dots refer to 2D-PIC laser-driven sources with 0.2, 0.6 and 2.0 μm substrate thicknesses, respectively; the cross markers are obtained with 3D-PIC laser-driven protons

We report the spectra obtained from simulations performed with 2.5 and 5.5 MeV monoenergetic protons in the left part of Fig. 3(a). They are normalized to the proton charge exploited in the simulations. In the right part of the figure, we also show a couple of simulated spectra collected with laser-driven protons (from the 0.2 μm thick bare target and the corresponding DLT with 4 μm thick near-critical layer). Both in the case of monoenergetic and laser-driven protons, the characteristic signals of all trace elements are present.

To obtain the number of emitted X-rays per unit of time, the X-ray yields for the various elements $N_{x} / N_{p}$ (i.e. the counts subtended by each peak in the spectra) must be multiplied by the average proton current *I* at the sample. As far as particle accelerators are concerned, we consider a reasonable range of $I = 1 - 10$ nA [40–42]. The resulting intervals of $\dot{N}_{x,i}$ for conventional accelerators are reported as blue areas in the panels of Fig. 3(b) for the various elements. All other points are associated with laser-driven PIXE results obtained by coupling 2D-PIC and MC simulations. They are expressed as $\dot{N}_{x,i} = N_{x,i} / N_{p} \cdot RR$, where $N_{p}$ is the number of protons per laser shot in Table 1 and $RR = 1$ Hz is laser repetition rate. The $\dot{N}_{x,i}$ values lying on one curve are obtained with the same target substrate and different near-critical layer thicknesses. For the minimum values of $\bar{E}_{p}$ (i.e. simple bare targets without near-critical layers), $\dot{N}_{x,i}$ strongly increases reducing the target thickness for all the detectable elements. However, values achieved with the bare targets are $1-2$ orders of magnitude smaller than those achieved with conventional accelerators even exploiting the 200 nm thick Al target.

As shown in the previous section, DLTs allow a strong increase in the laser-driven proton energies and numbers. As a result, the number of emitted X-rays per unit of time further increases as well. Notably, $\dot{N}_{x,i}$ obtained with DLTs are comparable to those from 1−10 nA current conventional accelerators. These results suggest that DLTs are required to achieve comparable performances with conventional accelerators, in terms of the number of characteristic X-rays. Moreover, all $\dot{N}_{x,i}$ obtained with DLTs and 2D-PIC simulations are close to each other for the various elements, indicating a mild dependence on both the substrate and near-critical layer thicknesses, thus on the proton cut-off energy. This could be an advantage in terms of the stability of the laser-driven source for PIXE analysis with DLTs.

As shown in the previous section the optimal DLT configuration for proton acceleration corresponds to the 200 nm substrate and 4 μm near-critical layer thicknesses. The same optimal condition stands also for laser-driven PIXE simulations carried out with 2D-PIC results. Therefore, we performed a laser-driven PIXE MC simulation using the realistic 3D-PIC proton energy spectrum. The results in terms of $\dot{N}_{x,i}$ are reported in Fig. 3(b) as cross markers. Even if $\bar{E}_{p}$ is lower compared to the equivalent 2D-PIC simulation result, the emitted number of X-rays is still comparable. Indeed, the lower average energy is counterbalanced by the higher number of accelerated protons per shot. From this result, we can conclude that nano-structured carbon foam-based DLTs can make laser-driven PIXE with compact lasers competitive with conventional accelerators for environmental sample analysis.

## Conclusions

Laser-driven particle accelerators are considered a promising alternative to conventional machines because of their potential cost-effectiveness and compactness. In particular, the use of advanced targets represents a robust solution to provide ion bunches in the 1−10 MeV energy range with commercial lasers. In this work, we have shown that near-critical DLTs which can be entirely produced via PVD techniques provide a significant enhancement of particle energy and number compared to standard target configurations. Also, precise control of DLT properties allows fine-tuning of the features of the accelerated ions. We demonstrated with PIC and MC simulations that a suitable choice of the DLT parameters, specifically the thickness of both the near-critical layer and the substrate, enables the exploitation of compact, 20 TW lasers for applications of strategic societal relevance such as pollution monitoring via the PIXE technique. Moreover, combining DLTs and the laser system considered in this work could enable other applications based on secondary radiation like neutron imaging and radiography. The performance of the laser-driven accelerator here proposed is comparable to standard accelerators, thus effectively showing how double-layer targets have a potentially far-reaching impact on both fundamental research and sustainable development.

## Data Availability

The datasets used and/or analysed during the current study are available from the corresponding author on reasonable request.

## References

[1] Bolton P, Parodi K, Schreiber J (2018). Applications of laser-driven particle acceleration.

[2] Macchi A, Borghesi M, Passoni M (2013). Ion acceleration by superintense laser-plasma interaction. Rev Mod Phys.

[3] Barberio M, Veltri S, Scisciò M, Antici P (2017). Laser-accelerated proton beams as diagnostics for cultural heritage. Sci Rep.

[4] Passoni M, Fedeli L, Mirani F (2019). Superintense laser-driven ion beam analysis. Sci Rep.

[5] Mirani F, Maffini A, Casamichiela F, Pazzaglia A, Formenti A, Dellasega D, Russo V, Vavassori D, Bortot D, Huault M (2021). Integrated quantitative pixe analysis and edx spectroscopy using a laser-driven particle source. Sci Adv.

[6] Puyuelo-Valdes P, Vallières S, Salvadori M, Fourmaux S, Payeur S, Kieffer J-C, Hannachi F, Antici P (2021). Combined laser-based x-ray fluorescence and particle-induced x-ray emission for versatile multi-element analysis. Sci Rep.

[7] Verma HR (2007). Atomic and nuclear analytical methods.

[8] Mirani F, Maffini A, Passoni M (2023). Laser-driven neutron generation with near-critical targets and application to materials characterization. Phys Rev Appl.

[9] Mirani F, Calzolari D, Formenti A, Passoni M (2021). Superintense laser-driven photon activation analysis. Commun Phys.

[10] Passoni M, Arioli F, Cialfi L, Dellasega D, Fedeli L, Formenti A, Giovannelli AC, Maffini A, Mirani F, Pazzaglia A (2019). Advanced laser-driven ion sources and their applications in materials and nuclear science. Plasma Phys Control Fusion.

[11] Prencipe I, Metzkes-Ng J, Pazzaglia A, Bernert C, Dellasega D, Fedeli L, Formenti A, Garten M, Kluge T, Kraft S, Garcia AL, Maffini A, Obst-Huebl L, Rehwald M, Sobiella M, Zeil K, Schramm U, Cowan TE, Passoni M (2021). Efficient laser-driven proton and bremsstrahlung generation from cluster-assembled foam targets. New J Phys.

[12] Maffini A, Orecchia D, Pazzaglia A, Zavelani-Rossi M, Passoni M (2022). Pulsed laser deposition of carbon nanofoam. Appl Surf Sci.

[13] Pazzaglia A, Fedeli L, Formenti A, Maffini A, Passoni M (2020). A theoretical model of laser-driven ion acceleration from near-critical double-layer targets. Commun Phys.

[14] Maffini A, Pazzaglia A, Dellasega D, Russo V, Passoni M (2019). Growth dynamics of pulsed laser deposited nanofoams. Phys Rev Mater.

[15] Maffini A, Pazzaglia A, Dellasega D, Russo V, Passoni M (2022). Production of carbon nanofoam by pulsed laser deposition on flexible substrates. Nanoporous carbons for soft and flexible energy devices.

[16] Prencipe I, Fuchs J, Pascarelli S, Schumacher D, Stephens R, Alexander N, Briggs R, Büscher M, Cernaianu M, Choukourov A (2017). Targets for high repetition rate laser facilities: needs, challenges and perspectives. High Power Laser Sci Eng.

[17] Flacco A, Sylla F, Veltcheva M, Carrié M, Nuter R, Lefebvre E, Batani D, Malka V (2010). Dependence on pulse duration and foil thickness in high-contrast-laser proton acceleration. Phys Rev E.

[18] Ceccotti T, Lévy A, Popescu H, Réau F, d’Oliveira P, Monot P, Geindre J, Lefebvre E, Martin P (2007). Proton acceleration with high-intensity ultrahigh-contrast laser pulses. Phys Rev Lett.

[19] Poole PL, Obst L, Cochran GE, Metzkes J, Schlenvoigt H-P, Prencipe I, Kluge T, Cowan T, Schramm U, Schumacher DW (2018). Laser-driven ion acceleration via target normal sheath acceleration in the relativistic transparency regime. New J Phys.

[20] Boivin F, Vallières S, Fourmaux S, Payeur S, Antici P (2022). Quantitative laser-based x-ray fluorescence and particle-induced x-ray emission. New J Phys.

[21] Sarakinos K, Alami J, Konstantinidis S (2010). High power pulsed magnetron sputtering: a review on scientific and engineering state of the art. Surf Coat Technol.

[22] Dellasega D, Mirani F, Vavassori D, Conti C, Passoni M (2021). Role of energetic ions in the growth of fcc and *ω* crystalline phases in ti films deposited by hipims. Appl Surf Sci.

[23] Vavassori D, Mirani F, Gatti F, Dellasega D, Passoni M (2023). Role of magnetic field and bias configuration on hipims deposition of w films. Surf Coat Technol.

[24] Zeil K, Kraft S, Bock S, Bussmann M, Cowan T, Kluge T, Metzkes J, Richter T, Sauerbrey R, Schramm U (2010). The scaling of proton energies in ultrashort pulse laser plasma acceleration. New J Phys.

[25] Miyamoto Y, Fujii Y, Yamano M, Harigai T, Suda Y, Takikawa H, Kawano T, Nishiuchi M, Sakaki H, Kondo K (2016). Preparation of self-supporting au thin films on perforated substrate by releasing from water-soluble sacrificial layer. Jpn J Appl Phys.

[26] Banerjee A, Banerjee S (2016). Growing gold fractal nano-structures and studying changes in their morphology as a function of film growth rate. Mater Res Express.

[27] Fedeli L, Huebl A, Boillod-Cerneux F, Clark T, Gott K, Hillairet C, Jaure S, Leblanc A, Lehe R, Myers A, Piechurski C, Sato M, Zaim N, Zhang W, Vay J-L, Vincenti H (2022). Pushing the frontier in the design of laser-based electron accelerators with groundbreaking mesh-refined particle-in-cell simulations on exascale-class supercomputers. SC22: international conference for high performance computing, networking, storage and analysis.

[28] Derouillat J, Beck A, Pérez F, Vinci T, Chiaramello M, Grassi A, Flé M, Bouchard G, Plotnikov I, Aunai N, Dargent J, Riconda C, Grech M (2018). Smilei: a collaborative, open-source, multi-purpose particle-in-cell code for plasma simulation. Comput Phys Commun.

[29] Liu J-L, Chen M, Zheng J, Sheng Z-M, Liu C-S (2013). Three dimensional effects on proton acceleration by intense laser solid target interaction. Phys Plasmas.

[30] Xiao KD, Zhou CT, Jiang K, Yang YC, Li R, Zhang H, Qiao B, Huang TW, Cao JM, Cai TX, Yu MY, Ruan SC, He XT (2018). Multidimensional effects on proton acceleration using high-power intense laser pulses. Phys Plasmas.

[31] Sgattoni A, Londrillo P, Macchi A, Passoni M (2012). Laser ion acceleration using a solid target coupled with a low-density layer. Phys Rev E.

[32] Sinigardi S, Babaei J, Turchetti G (2018). TNSA proton maximum energy laws for 2d and 3d PIC simulations. Nucl Instrum Methods Phys Res, Sect A, Accel Spectrom Detect Assoc Equip.

[33] Babaei J, Gizzi LA, Londrillo P, Mirzanejad S, Rovelli T, Sinigardi S, Turchetti G (2017). Rise time of proton cut-off energy in 2d and 3d PIC simulations. Phys Plasmas.

[34] Vallières S, Salvadori M, Permogorov A, Cantono G, Svendsen K, Chen Z, Sun S, Consoli F, d’Humières E, Wahlström C-G (2021). Enhanced laser-driven proton acceleration using nanowire targets. Sci Rep.

[35] Neely D, Foster P, Robinson A, Lindau F, Lundh O, Persson A, Wahlström C-G, McKenna P (2006). Enhanced proton beams from ultrathin targets driven by high contrast laser pulses. Appl Phys Lett.

[36] Prasad R, Andreev AA, Ter-Avetisyan S, Doria D, Quinn KE, Romagnani L, Brenner CM, Carroll DC, Dover NP, Neely D, Foster PS, Gallegos P, Green JS, McKenna P, Najmudin Z, Palmer CAJ, Schreiber J, Streeter MJV, Tresca O, Zepf M, Borghesi M (2011). Fast ion acceleration from thin foils irradiated by ultra-high intensity, ultra-high contrast laser pulses. Appl Phys Lett.

[37] Fedeli L, Formenti A, Cialfi L, Pazzaglia A, Passoni M (2018). Ultra-intense laser interaction with nanostructured near-critical plasmas. Sci Rep.

[38] Fedeli L, Formenti A, Bottani CE, Passoni M (2017). Parametric investigation of laser interaction with uniform and nanostructured near-critical plasmas. Eur Phys J D.

[39] Allison J, Amako K, Apostolakis J, Arce P, Asai M, Aso T, Bagli E, Bagulya A, Banerjee S, Barrand G (2016). Recent developments in geant4. Nucl Instrum Methods Phys Res, Sect A, Accel Spectrom Detect Assoc Equip.

[40] Sa’adeh H, Chiari M (2019). Atmospheric aerosol analysis at the pixe–rbs beamline in the university of Jordan van de Graaff accelerator (juvac). X-Ray Spectrom.

[41] Fagbenro A, Yinusa T, Ajekiigbe K, Oke A, Obiajunwa E (2021). Assessment of heavy metal pollution in soil samples from a gold mining area in osun state, Nigeria using proton-induced x-ray emission. Sci Afr.

[42] Bandhu H, Puri S, Garg M, Singh B, Shahi J, Mehta D, Swietlicki E, Dhawan D, Mangal P, Singh N (2000). Elemental composition and sources of air pollution in the city of chandigarh, India, using edxrf and pixe techniques. Nucl Instrum Methods Phys Res, Sect B, Beam Interact Mater Atoms.

[43] Mantero A, Ben Abdelouahed H, Champion C, El Bitar Z, Francis Z, Guèye P, Incerti S, Ivanchenko V, Maire M (2011). Pixe simulation in geant4. X-Ray Spectrom.

